# Effect of a common missense variant in LIPA gene on fatty liver disease and lipid phenotype: New perspectives from a single‐center observational study

**DOI:** 10.1002/prp2.820

**Published:** 2021-09-02

**Authors:** Andrea Pasta, Paolo Borro, Anna Laura Cremonini, Elena Formisano, Giulia Tozzi, Stefano Cecchi, Raffaele Fresa, Sara Labanca, Afscin Djahandideh, Samir Giuseppe Sukkar, Antonino Picciotto, Livia Pisciotta

**Affiliations:** ^1^ Department of Internal Medicine University of Genoa Genoa Italy; ^2^ Gastroenterology Unit Department of Internal Medicine IRCCS Ospedale Policlinico San Martino University of Genoa Genoa Italy; ^3^ Dietetics and Clinical Nutrition Unit IRCCS Ospedale Policlinico San Martino Genoa Italy; ^4^ Nutritional Unit ASL‐1 Imperiese Giovanni Borea Civil Hospital Sanremo Italy; ^5^ Division of Metabolism and Research Unit of Metabolic Biochemistry Department of Pediatrics IRCCS Bambino Gesù Children's Hospital Rome Italy; ^6^ Hepatology, Gastroenterology and Nutrition Unit IRCCS "Bambino Gesù" Children's Hospital Rome Italy

**Keywords:** controlled attenuation parameter, hepatic steatosis, *LIPA* gene, NAFLD, rs1051338

## Abstract

Lysosomal acid lipase deficiency (LAL‐D) is an autosomal recessive disease characterized by hypoalphalipoproteinemia, mixed hyperlipemia, and fatty liver (FL) due to mutations in *LIPA*se A, lysosomal acid type (*LIPA*) gene. The rs1051338 single‐nucleotide polymorphism (SNP) in *LIPA* gene, in vitro, could adversely affect the LAL activity (LAL‐A). Nonalcoholic fatty liver disease (NAFLD) is often associated with metabolic syndrome, and the diagnosis requires the exclusion of excess of alcohol intake and other causes of hepatic disease. The aim of the study was to evaluate the impact of rs1051338 rare allele on lipid phenotype, severity of FL, and LAL‐A in patients suffering from dyslipidemia associated with NAFLD. We selected 74 subjects with hypoalphalipoproteinemia or mixed hyperlipemia and evaluated transaminases, liver assessment with controlled attenuation parameter (CAP), LAL‐A, rs1051338 SNP genotype. The presence of rare allele caused higher levels of triglycerides and hepatic transaminase and lower levels of high‐density lipoprotein cholesterol (HDL‐C). Multivariate analysis highlighted independent association between rare allele and FL severity in subjects with NAFLD. The rs1051338 SNP may modulate FL severity and atherogenic dyslipidemia in patients suffering from NAFLD.

## Graphical abstract

A common polymorphic variant(rs1051338) in LIPA gene has been associated to atherogenic dyslipidemia,metabolic syndrome, obesity, and cardiovascular disease and, in vitro, couldadversely affect the LAL activity. Seventy‐four patients with metabolic riskfactors have been enrolled: higher levels oftriglycerides and hepatic transaminases, and lower levels of high‐densitylipoprotein cholesterol have been observed in patients with the rare allele ofrs1051338 in LIPA gene. The presence of rare polymorphic variant seems to beassociated with severe steatosis in subjects affected by NAFLD.

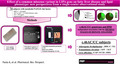

**Abbreviations**
ALT: alanine aminotransferase
AST: aspartate aminotransferase
BMI: body mass index
CAP: controlled attenuation parameter
dB/m: decibel/meter
DBS: dried blood spots
FL: fatty liver
HDL‐C: high‐density lipoprotein cholesterol
IQR: interquartile range
KPa: kilopascal
LAL‐A: lysosomal acid lipase activity
LAL‐D: lysosomal acid lipase deficiency
LDL‐C: low‐density lipoprotein cholesterol
lipase A: lysosomal acid type
MAF: minor allele frequency
NAFLD: nonalcoholic fatty liver disease
OR: odds ratio
SNP: single‐nucleotide polymorphism
SWE: shear wave elastography
TC: total cholesterol
TG: triglycerides

## 1 INTRODUCTION

Lipase A, lysosomal acid type (*LIPA*) gene encodes for lysosomal acid *LIPA*se (LAL), an enzyme that is able to catalyze triglycerides (TG) and cholesterol esters hydrolysis in lysosomes. Lysosomal acid *LIPA*se deficiency (LAL‐D) is an autosomal recessive disease characterized by hypoalphalipoproteinemia, mixed hyperlipemia and hepatic steatosis.^1^, ^2^

The rs1051338 polymorphism is a common variant of the *LIPA* gene with a global frequency of the minor allele (GMAF) of 0.25010. The variant is located at the exon 2 level and involves the replacement of adenine with a cytosine in position 46 (ACC>CCC). A recent in silico and ex‐vivo study^3^ has reported that this nucleobases substitution causes an aminoacid change at a translational level: 16‐threonine (Thr, T) with 16‐proline (Pro, P) at the level of the signal peptide of the protein. Consequently, the signal peptide of LAL‐Pro is reduced of two aminoacids compared with that of LAL‐Thr, causing an early truncation of the central core and a displacement of two amino acids at the cleavage site. As a result, the change in the signal peptide of LAL disrupts the normal sorting and transport of LAL to the lysosome. The polymorphic LAL is susceptible to cytosolic proteasomal degradation, thus reducing lysosomal LAL protein and activity (LAL‐A).^3^ Moreover, the rs1051338 polymorphism in *LIPA* gene has been previously associated by disequilibrium linkage to increased risk of atherogenic dyslipidemia, metabolic syndrome, obesity, and cardiovascular disease.^3^, ^4^

Non‐alcoholic fatty liver disease (NAFLD) is the presence of fat in the liver (FL) after the exclusion of alcohol excess or other causes of liver disease: NAFLD pathogenesis is multifactorial and influenced by presence of metabolic syndrome or genetic polymorphisms.^5^ LAL‐A reduction was observed in patients with NAFLD^6^ and, in the metabolic‐molecular prospective, LAL impairment results in reduced hepatocyte effectiveness in processing lipids, which, consequently, are accumulated in the liver.^1^ The rare allele (c. 46C, p.16P) of rs1051338 has been previously associated with LAL‐A slight deficiency and could be linked to NAFLD pathogenesis.

Therefore, the LAL‐A reduction causes the activation of the transcription factors of the sterol regulatory element‐binding protein (SREBP) family with consequent activation of lipogenesis which results in an alteration of the release of cholesterol and free fatty acids in the cytosol.^7^ This mechanism supports the increase in intracellular fatty acids causing the development and progression of NAFLD.^8^ On the other hand, the cytosolic accumulation of fatty acids and triglycerides typical of NAFLD can cause a reduction of LAL‐A by a negative feedback regulation.^9^

The clinical characterization of the rs1051338 polymorphism can provide useful prognostic data as well as a better diagnostic classification. The rs1051338 polymorphism may be consider as other polymorphisms whose association with NAFLD was already established.^10^, ^11^ The study of genetic background of fatty liver and NAFLD could lead to better therapeutic strategies soon.

We suggest the hypothesis that the presence of rs1051338 polymorphism may exacerbate the pathological phenotype of patients suffering from dyslipidemia and hepatic steatosis associated with NAFLD. Thus, the aim of the study was to evaluate the impact of rs1051338 rare allele on lipid phenotype, severity of FL, and LAL‐A in patients suffering from dyslipidemia associated with NAFLD.

## 2 MATERIALS AND METHODS

### 2.1 Study design

The present is an observational study designed to test the influence of the rs1051338 polymorphism in *LIPA* gene on lipid phenotype, liver assessment, and LAL‐A.

Inclusion criteria were established to screen low‐moderate LAL‐D and were obtained by an adaptation of a model described in the literature,^12^ which were originally designed to identify genetic‐determined LAL‐D. Low‐density lipoprotein cholesterol levels were not considered as inclusion criteria to avoid the risk of underestimation due to the presence of high TG level typical of mixed LAL‐D‐like dislipidemia. Conversely, low HDL‐C levels were used as a main inclusion criterion because they are strongly associated with LAL‐D.^2^, ^13^, ^14^, ^15^, ^16^, ^17^

Thus, patients included had high‐density lipoprotein cholesterol (HDL‐C) <50 mg/dl and, at least, one of the following characteristics: alanine aminotransferase (ALT) or aspartate aminotransferase (AST) >1.5 maximum laboratory value or TG >150 mg/dl or hepatomegaly or hepatic steatosis in the context of NAFLD.

Exclusion criteria were age <18 years and >80 years, acute or chronic artery or heart disease, chronic and acute liver disease with different diagnosis than NAFLD, acute and chronic nephropathies, presence of acute and chronic infections (including HIV), active malignant neoplasms, uncontrolled endocrinopathies, inflammatory bowel diseases, chronic therapy with hepatotoxic drugs, pregnancy, LAL‐A levels <0.15 nmol/spot/h, and use of lipid lowering treatments.

All included patients were screened in the outpatient section of the Lipid Clinic of IRCCS Policlinic San Martino Hospital, University of Genoa, Italy, and who met inclusion criteria were enrolled consecutively.

At inclusion visit, anamnestic and anthropometric data (weight, height, body mass index, blood pressure, and heart rate) were collected and all subjects underwent physician examination. Blood tests performed in authorized laboratory within 4 weeks since baseline were evaluated and total cholesterol (TC), HDL‐C and TG, AST and ALT were registered. LDL‐C was calculated by the Friedewald formula. Blood samples were obtained and stored in ethylene‐di‐amino‐tetra‐acetic acid K3 to perform DNA extraction, analyze genetic polymorphism, and determine LAL‐A on dried blood spots (DBS). Thus, all patients underwent ultrasound liver assessment for the evaluation of steatosis and liver stiffness.

The study (2020‐383 id.10748) was approved by the Institutional Review Board of IRCCS Ospedale Policlinico San Martino (Genoa, Italy) and conducted in accordance with the guidelines of the Declaration of Helsinki. All patients signed an informed consent form before inclusion in the present study.

### 2.2 Genetic analysis

Genomic DNA was extracted from peripheral blood leukocytes with the standard techniques.^18^ Genetic analysis was performed by restriction fragment length polymorphism analysis for rs1051338 of *LIPA* gene which was previously described.^19^

### 2.3 LAL activity determination

Blood LAL activity (nmol/spot/h) was measured with DBS extracts using the inhibitors Lalistat2. Peripheral blood was collected with ethylene‐diamine‐tetra acetic acid blood, spotted on to filter paper, and allowed to dry overnight at room temperature. Samples were stored double‐bagged with desiccant at −20°C and analyzed within 2 weeks of storage. Uninhibited and inhibited with Lalistat 2 (Chemical Tools, South Bend, IN, USA) activities were dosed. LAL activity was determined by subtracting activity in the inhibited reaction from uninhibited reaction (total lipase) and expressed as nmol/spot/h of methylumbelliferone.^20^ Inter and intra‐assay variations were 2.4 and 2.3%, respectively.

### 2.4 Liver assessment and results ranking

Liver evaluation was performed by a single expert operator (PB) using the multi‐purpose LOGIQ S8 XDclear 2.0 system (GE Healthcare, Via Galeno, 36–20126 Milano). Liver steatosis was assessed by FibroScan® (Echosens, Paris, France) Controlled Attenuation Parameter (CAP), while fibrosis, as liver stiffness (LS), was measured with two different techniques: FibroScan® Vibration‐Controlled Transient Elastography (VCTE) and Two‐dimensional shear wave elastography (SWE). Scientific bases of CAP, VCTE, and SWE were described elsewhere.^21^, ^22^, ^23^, ^24^

All patients undergone liver examination were fasting at least from 6 hours. CAP and VCTE were performed by the FibroScan® system, and values were expressed as median and inter quartile range/median (IQR/median) and measure units were decibel/meter (dB/m) and kilopascal (KPa), respectively. SWE were assessed by a software included in the LOGIQ S8 XDclear 2.0 system using the convex ultrasound probe and values were expressed as median in KPa and IQR/median. Final values were computed only when valid measures were up to 10 and acceptability criteria were IQR/median <30% and success rate >60%.^25^ According with the literature,^26^, ^27^, ^28^ in Table S1, CAP, VCTE, and SWE are reported the cutoff values used for steatosis and fibrosis staging.

Patients were also divided depending on early versus advanced stages of steatosis as follow: S0, S1, and S2 were considered early stage, patients with S3 were considered at severe stage of disease.^29^

### 2.5 Statistical analysis

Statistical analysis was performed using IBM SPSS Statistics version 25, Release Version 25.0; SPSS, Inc., 2017, Chicago, IL, www.spss.com).

An ad hoc statistical analysis was performed to estimate the minimum sample size of the study. Steatosis ranking method returned an ordinal variable (S) between 0 and 3 (S0, S1, S2, S3), thus ordinal regression model was chosen as a statistical test. Assumptions were rs1051338 minor allele frequency (MAF) and a mean difference of 10% in patients’ distribution within steatosis groups. According to literature,^30^ minimum sample size of 70 patients for 2.0‐fold cumulative odd ratio (OR) and 62 patients for 2.5‐fold cumulative OR. The recruitment target was set over these values.

Kolmogorov–Smirnov analysis was performed to test the normality of continuous variables. Results of continuous variables were expressed as median and IQR. For ordinal and nominal variables, contingency tables were used indicating frequency and percentage in the population. For the comparison of continuous variables between different patients’ groups, nonparametric tests of Kruskal–Wallis or Mann–Whitney were used when appropriate. Nominal variables were examined with the Pearson chi square (x2) test and continuous variables with Spearman's rank correlation index.

Multivariate ordinal regression was used for testing risk factors, nominal and continuous (these last ranked in quartiles in Table S2) influence on outcomes using classic and stepwise methods. Thus, ordinal multivariate analysis was performed on steatosis severity groups (ranked as 0 for S0, 1 for S1, 2 for S2, and 3 for S3) and in the former analysis, the covariates were age, sex, TG, body mass index (BMI), and rs1051338 genotypes. The covariates of ordinal multivariate analysis for fibrosis stages were steatosis severity, age, sex, TG, BMI, and rs1051338 genotypes.

Multivariate logistic regression was also performed to test predictors of severe stage of disease. Data analyzed were set as follow: 0 for presence of S0, S1 and S2 and 1 for presence of S3, 1 for male and 0 for female sex. BMI, TG levels and age were ranked in quartiles (Table S2).

## 3 RESULTS

At inclusion visit, a total of 74 subjects met inclusion criteria and were enrolled in the study (57 males and aged between 30 and 77 years). Table 1 gives demographic, anthropometric, hematologic, and anamnestic characteristics of all patients and summarizes the results of rs1051338 genetic analysis and LAL‐A. Ultrasound results and prevalence of steatosis and fibrosis ranked as described in methods are still reported in Table 1. Only 5 out of 74 patients suffer from type 2 diabetes mellitus, but the levels of glycated hemoglobin were between 44 and 52 mmol/mol. Most patients enrolled (n. 65 out of 74, 87.8%) were affected by hepatic steatosis, while 41 (55.4%) had advanced disease or severe steatosis. Data regarding pharmacological therapy have been reported in Table S3.

**TABLE 1:** Sample general characteristics

Parameter	Value
Age [years: mean ± SD; median; IQR]	55 ± 11; 56 (48–63)
Sex [F/M: n; %]	17 (23,0%) / 57 (77,0%)
Type 2 Diabetes Mellitus [absence/presence: n, %]	69 (93,2%) / 5 (6,8%)
BMI [Kg/m^2^: mean ± SD; median; IQR]	28,6 ± 3,7; 28,1 (26,3–30,5)
TC [mg/dL: mean ± SD; median; IQR]	237 ± 59; 233 (202–268)
HDL‐C [mg/dL: mean ± SD; median; IQR]	39 ± 7; 39 (33–44)
LDL‐C [mg/dL: mean ± SD; median; IQR]	157 ± 61; 156 (115–183)
TG [mg/dL: mean ± SD; median; IQR]	339 ± 283; 258 (160–394)
Glycemia [mg/dL: mean ± SD; median; IQR]	98 ± 15; 95 (88–104)
AST [UI/L: mean ± SD; median; IQR]	30 ± 11; 26 (22–36)
ALT [UI/L: mean ± SD; median; IQR]	40 ± 19; 36 (23–54)
rs1051338 [n; %]	
c.46AA p.16TT	40 (54,1%)
c.46AC p.16TP	29 (39,2%)
c.46CC p.16PP	5 (6,8%)
LAL‐A [mmol/spot/h: mean ± SD; median; IQR]	0,51 ± 0,21; 0,45 (0,37–0,62)
SWE [kPa: mean ± SD; median; IQR]	5,72 ± 1,29; 5,67 (5,00–6,25)
CAP [dB/m: mean ± SD; median; IQR]	297 ± 62; 308 (269–345)
VCTE [kPa: mean ± SD; median; IQR]	6,06 ± 2,19; 5,79 (4,54–6,60)
Steatosis Severity [n; %]	
S0	9 (12,2%)
S1	9 (12,2%)
S2	15 (20,3%)
S3	41 (55,4%)

Fibrosis Severity [n; %]	SWE	VCTE
F0	58 (78,4%)	61 (82,4%)
F1	3 (4,1%)	
F2	10 (13,5%)	6 (8,1%)
F3	1 (1,4%)	3 (4,1%)
F4	2 (2,7%)	4 (5,4%)

Abbreviations: BMI, body mass index; CAP, controlled attenuation parameter; F, female; h, hours; LAL‐A, Lysosomal acid Lipase activity; M, male; SWE, Two‐dimensional shear wave elastography; VCTE, vibration‐controlled transient elastography.

**FIGURE 1:**
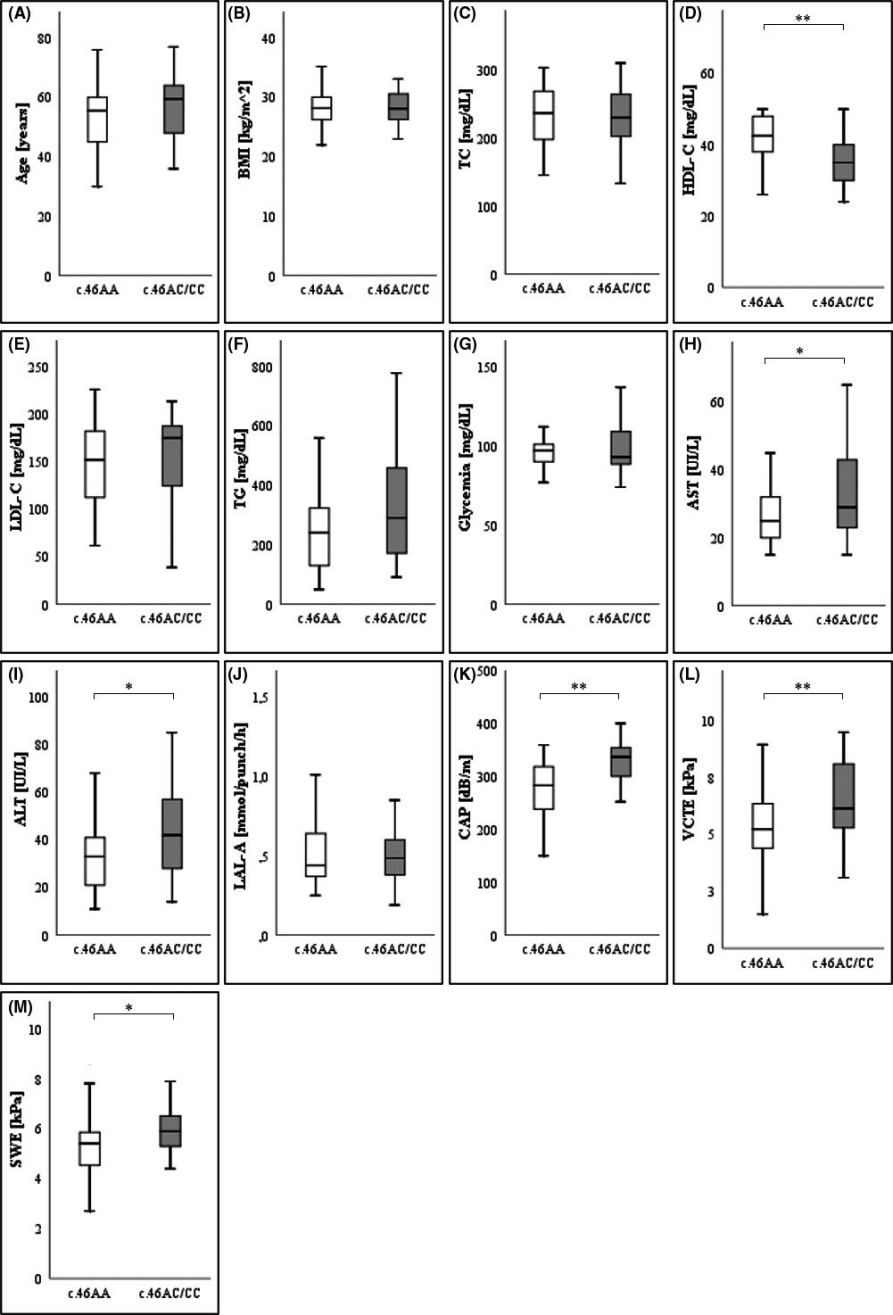
(A) Age, (B) BMI, (C) TC, (D) HDL‐C, (E) LDL‐C, (F) TG, (G) Glycemia, (H) AST, (I) ALT, (L) LAL‐A, (M) CAP, (N) VCTE,and (O) SWE levels in different rs1051338 SNP genotypes. BMI, Body mass index; LAL‐A, Lysosomal acid lipase activity; SWE, Two‐dimensional shear wave elastography; CAP, Controlled attenuation parameter; VCTE, FibroScan® Vibration‐controlled transient elastography. **p < .01 for Wilcoxon–Mann–Whitney test. *p < .05 for Wilcoxon–Mann–Whitney test

Overall patients carrying the rare allele (c.46AC or c.46CC) were 34 (46%), while control subjects (c.46AA) were 40 (54%). The levels of AST, ALT, CAP, VCTE, and SWE were significantly higher in c.46AC/CC subjects compared with c.46AA patients. Conversely, the latter (c.46AA patients) had higher HDL‐C levels than those of carriers of rare allele (Figure 1). TG levels were higher in patients with c.46AC or c.46CC genotypes than those carried the common allele, but the difference did not reach the statistical significance (*p* = 0.075).

Furthermore, significant differences emerged in steatosis severity (S0, S1, S2, S3) within genotypic groups: the rare allele (c.46C) was more frequent in patients with more severe steatosis and vice versa (Table 2). No other significant differences were found in other considered variables between c.46C and c.46AA genotypes (Figure 1 and Table 2).

**TABLE 2:** Demographic, anamnestic and liver ultrasonographic assessment in different rs1051338 genotypes

Parameter	c.46AA	c.46AC/CC	*p*‐value*
Sex [F/M: n; %]	8 (20,0%) / 32 (80,0%)	9 (26,5%) / 25 (73,5%)	.435
Type 2 Diabetes Mellitus [absence/presence: n, %]	38 (95,0%) / 2 (5,0%)	31 (91,2%) / 3 (8,8%)	.426
Steatosis Severity [n; %]			
S0	8 (20,0%)	1 (2,9%)	.**016**
S1	7 (17,5%)	2 (5,9%)	
S2	9 (22,5%)	6 (17,6%)	
S3	16 (40,0%)	25 (73,5%)	

Fibrosis Severity [n; %]	SWE	VCTE	SWE	VCTE	*p*‐value*
F0	34 (85,0%)	37 (92,5%)	24 (70,6%)	24 (70,6%)	.380** .062***
F1	1 (2,5%)		2 (5,9%)		
F2	3 (7,5%)	2 (5,0%)	7 (20,6%)	4 (11,8%)	
F3	1 (2,5%)	1 (2,5%)	0 (0,0%)	2 (5,9%)	
F4	1 (2,5%)	0 (0,0%)	1 (2,9%)	4 (11,8%)	

*Note*: Bold values denote statistical significance at the *p* < .05 level. Abbreviations: CAP, controlled attenuation parameter; F, female; M, male; *p*, *p*‐value; SWE, Two‐dimensional shear wave elastography; VCTE, vibration‐controlled transient elastography.

* Pearson's chi‐squared test

** *p*‐value for difference in SWE

*** *p*‐value for difference in VCTE.

No statistically significant differences emerged within steatosis severity groups (S) (Table S4), except for what was reported for the rs1051338 polymorphism in Table 2 and median BMI (25.7 IQR 23.9–26.2 for S1, 27.1, IQR 25.0–28.7 for S2 and 29.8, IQR 27.7–31.8 for S3 with *p* = .049 between S1 and S3 and *p* = .02 between S2 and S3).

Higher severity fibrosis groups (VCTE measured) were associated with higher TG, AST, and BMI levels, and lower HDL‐C levels than healthier patients (Table S5 and S6). Moreover, rare allele (c.46C) was borderline (*p* = 0.062) more frequently associated with severe fibrosis at VCTE (Table S6). No statistically significant differences emerged within fibrosis severity groups (F) when assessed with SWE, except for the age of patients, see Table S5.

**FIGURE 2:**
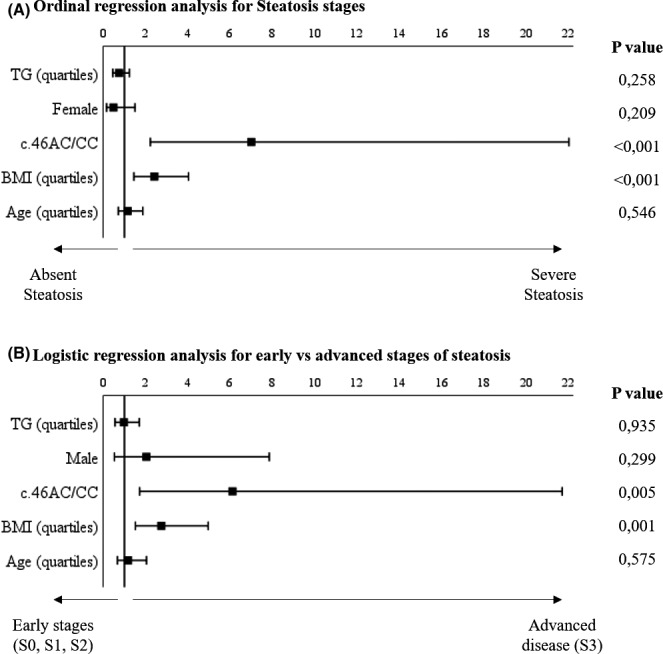
Forrest‐Plot of the multivariate (A) ordinal regression analysis for the different stages of steatosis and (B) logistic regression analysis S0, 1, 2 vs S3. BMI, Body Mass Index; F, female; *p*, *p*‐value

Steatosis severity was independently associated with the rare allele (c.46C) and increasing of BMI in ordinal regression analysis (Figure 2A). The analysis performed for hepatic fibrosis measured with VCTE showed that higher stages of fibrosis were associated with higher TG and BMI levels and to male gender (Figure S1A). No statistically independent variables were correlated to fibrosis staged with SWE (Figure S1B).

Finally, the logistic regression analysis in Figure 2B shows statistically significant association between the risk allele (c.46C) and the presence of severe steatosis (S3) in contrast to early stages of disease (S0, S1, and S2). Additionally, the higher BMI was independently correlated with the severe stage (S3) of steatosis.

## 4 DISCUSSION

In this study, the association between the rs1051338 SNP in the *LIPA* gene and hepatic steatosis was evaluated in patients with atherogenic dyslipidemia and NAFLD. Inclusion criteria used for patients’ enrollment and designed for identification of mild–moderate LAL‐D were effective in producing homogeneous sample and limited variability range of age, BMI, and lipid and glycemic profile.

The prevalence of rare allele (c.46C) rs1051338 SNP was observed in 46% of cases, 39.2% HZ and 6.8% OZ, with MAF (minor allele frequency) of 26.4%: this distribution respects the Hardy–Weinberg equilibrium and agreed with Global MAF of 25.010% reported in the literature. NAFLD was diagnosed in 87.8% of patients (55.4% S3 or severe steatosis) which represented a very high prevalence, although the literature reports that NAFLD prevalence ranged between 50 and 95% in subjects with metabolic risk factors and dyslipidemia, such as our sample.^31^, ^32^, ^33^

Lower HDL‐C and borderline higher TG levels than wild type were observed in the rare allele carriers. This findings agreed with the literature, but such differences have been observed only in linkage of disequilibrium study.^4^, ^34^ On the other hand, the finding of higher CAP, AST, and ALT values in c.46AC/CC patients compared with controls has been never observed. Furthermore, the presence of the rare polymorphism allele was independently associated with greater severity of hepatic steatosis (O.R. 7.0, IC 2.2–22.0 with *p* < .001) in ordinal multivariate analysis, achieving one of the primary endpoints of the study. In addition, the binary analysis performed with logistic regression showed that sever steatosis (i.e., S3) resulted independently associated with the c.46AC/CC genotypes (O.R. 6.1, IC 1.7–21.7 with *p* = .005) in contrast to lower stages of steatosis (i.e., S0, S1, and S2). Morris GE et al. ^3^ identified the possible loss of function effect of the rs1051338 SNP on LAL activity using *in silico* and *in vitro* tests. These latter results were consistent with our study and could support the existence of attenuated forms of LAL‐D with milder clinical features caused by the rs1051338 SNP compared with the classical autosomal recessive forms. Our data suggest that the presence of a polymorphic variant can contribute to the pathogenesis of the NAFLD by increasing the risk to develop a more severe phenotype of disease. On the contrary, the presence of rs1051338 is unlikely to be independently causative of NAFLD.

Finally, more severe fibrosis was associated with hypertriglyceridemia, high BMI, and male gender, in agreement with the literature.^35^

Unfortunately, no significant differences have been observed in LAL‐A values within rs1051338 genotypes (mean ± SD: c.46AC/CC 0.53 ± 0.23 vs c.46AA 0.50 ± 0.19 mmol/spot/h with *p* = .501). This results agreed with Morris GE et al.’s study^3^ in which the differences in LAL‐A were not reported in whole macrophages, instead lower LAL‐A levels were only measured directly in lysosome of the rare allele carriers. Unfortunately, LAL‐A in our study was not directly measured in lysosomes because the method was not technically available. In accordance with our observation, Morris Ge et al.^3^ reported that LAL‐A was not significantly different between carriers and noncarriers of the rs1051338 rare allele in whole cells, but the first ones have lower LAL‐A in lysosomal lysate. Our hypothesis is that the difference in LAL‐A is present between the two groups of patients at the lysosomal level; however, the method for evaluating LAL‐A in the lysosomal lysate was not available, and the measurement was performed only on the whole macrophage. Therefore, a main limitation of the present study was the lack of demonstration about the direct correlation between LAL‐A reduction and the presence of the rs1051338 allele‐risk in the *LIPA* gene. Moreover, LAL‐A measure on DBS had some limits highlighted in recent studies^36^, ^37^ and was originally designed for detecting very low LAL‐A levels (<0.15 nmol/spot/h).^12^

Other limitations of this study were the low sample size, and future studies need to be carried out to strengthen our preliminary findings. Thus, ad hoc analysis was conducted to estimate an adequate sample size and was based on the data available regarding the polymorphism of MAF and the incidence of hepatic steatosis in high metabolic‐risk patients. Furthermore, according to the ethics committee recommendation, the enrollment of patients suffered from difficulties due to the COVID‐19 pandemic was interrupted as sufficient statistical power was obtained.

The choice of cases and controls did not allow to demonstrate the causative effect of rs1051338 polymorphism on NAFLD, but to investigate whether patients carrying the rare allele and suffer from dyslipidemias or hepatic steatosis had an increased risk of a more severe phenotypes. As shown in the literature, the presence of a polymorphic variant can contribute to the pathogenesis of the disease, conversely a rare variant with a loss of function effect can be the main cause of a disease (i.e., Wolman disease).

The risk of misclassification is high using elastometric measures to assess hepatic steatosis, although the use of CAP has proven to be effective in defining the degree of steatosis. Shen et al.^29^ reported areas under the curves of 0.92, 0.92, and 0.88 for steatosis ≥5% (mild, ≥S1), ≥34% (moderate, ≥ S2), and ≥67% (severe, S3). Also, MRI or liver biopsy assessment of steatosis and liver fibrosis may had increase specificity and sensitivity of our measures. It would be useful to have data regarding the degree of lobular inflammation, however in Italy it is not possible to carry out liver biopsy outside the recommendations of good clinical practice, therefore, exclusively to clarify unknown or unclear diagnosis, to evaluate the prognosis, to improve therapeutic choices, or to perform specific histological examinations (i.e., immunohistochemical, immunological, others).

In conclusion, the rs1051338 polymorphism of the LIPA gene influences the lipid profile contributing to worse a form of dyslipidemia LAL‐D‐like and was significantly associated to a worse hepatic steatosis in subjects with NAFLD. These results should be confirmed by further studies, which could finally define rs1051338 functional nature and the existence of “mild” forms of LAL‐D that could play an important role in NAFLD pathogenesis. Furthermore, the elevation in transaminase levels observed in patients carrying the rare allele may suggest an increased risk of nonalcoholic steatohepatitis.

## 5 ETHICS APPROVAL STATEMENT AND PATIENT CONSENT STATEMENT

The study (2020‐383 id.10748) was approved by the Institutional Review Board of IRCCS Ospedale Policlinico San Martino (Genoa, Italy) and conducted in accordance with the guidelines of the Declaration of Helsinki. All patients signed an informed consent form before inclusion in the present study.

## CONFLICT OF INTEREST

The authors declare that there is no conflict of interest regarding the publication of this paper.

## AUTHOR'S CONTRIBUTION

Livia Pisciotta did conceptualization; Livia Pisciotta, Elena Formisano, Andrea Pasta, Anna Laura Cremonini, and Afscin Djahandideh performed data collection; Andrea Pasta: Formal analysis; Livia Pisciotta, Paolo Borro, Andrea Pasta, Elena Formisano, Anna Laura Cremonini, Sara Labanca, Samir Giuseppe Sukkar, and Afscin Djahandideh did Investigation; Livia Pisciotta, Giulia Tozzi, Stefano Cecchi, Raffaele Fresa, Paolo Borro and Andrea Pasta performed methodology; Livia Pisciotta did project administration; Livia Pisciotta and Antonino Picciotto did supervision; Andrea Pasta did Visualization; Andrea Pasta was involved in Roles/Writing—original draft; Livia Pisciotta, was involved in writing—review & editing.

## Supporting information

Supplementary Material

## Data Availability

The data that support the findings of this study are available from the corresponding author upon reasonable request.
