# Supplementary material for: Effect of a common missense variant in LIPA gene on fatty liver disease and lipid phenotype: New perspectives from a single‐center observational study
Source: Pharmacol Res Perspect. 2021 Sep 2;9(5):e00820. doi: 10.1002/prp2.820 (PMC8413903; doi:10.1002/prp2.820)
Supplement: Supplementary file 1 — Supplementary Material [file PRP2-9-e00820-s001.docx]

**Supplementary Material Index**

[**Supplementary Table S.1.** Steatosis and fibrosis severity raking groups according with methods 2](#_Toc71489686)

[**Supplementary Table S.2.** Quartiles of Age, BMI e triglycerides 3](#_Toc71489687)

[**Supplementary Table S.3.**  Pharmacological therapy used by patients 3](#_Toc71489688)

[**Supplementary Table S.4.** Demographic, anamnestic and biochemical assessment in different steatosis severity groups 4](#_Toc71489689)

[**Supplementary Table S.5.** Demographic, anamnestic and biochemical assessment in different fibrosis severity groups measured with SWE. 5](#_Toc71489690)

[**Supplementary Table S.6.** Demographic, anamnestic and biochemical assessment in different fibrosis severity groups measured with VCTE. 6](#_Toc71489691)

[**Supplementary Figure S.1.** Forrest-Plot of the multivariate ordinal regression analysis for the different stages of fibrosis measured with (A) VCTE and (B) SWE. 7](#_Toc71489692)

# **Supplementary Table S.1.** Steatosis and fibrosis severity raking groups according with methods

| **SEVERITY GROUPS** | **Cut-off Values** |
| --- | --- |
| **Steatosis (CAP)** |  |
| S0 | CAP<233.5 dB/m |
| S1 | 233.5≤CAP<268.5 dB/m |
| S2 | 268.5≤CAP<301.2 dB/m |
| S3 | CAP≥301.2 dB/m |
| **Fibrosis (SWE)** |  |
| F0 | SWE<6.48 kPa |
| F1 | 6.48≤SWE<6.60 |
| F2 | 6.60≤SWE<8.07 |
| F3 | 8.07≤SWE<9.31 |
| F4 | SWE≥9.31 |
| **Fibrosis (VTCE)** |  |
| F0-F1 | VTCE≤7 kPa |
| F2 | VTCE≤8.7 kPa |
| F3 | 8.7< VTCE≤10.3 kPa |
| F4 | VTCE>10.3 kPa |

SWE, Two-dimensional shear wave elastography; CAP, Controlled attenuation parameter; VTCE, FibroScan® Vibration-Controlled Transient Elastography;

# **Supplementary Table S.2.** Quartiles of Age, BMI e triglycerides

| **Variable** | **Quartiles** | | | |
| --- | --- | --- | --- | --- |
|  | **1^st^** | **2^nd^** | **3^rd^** | **4^th^** |
| Age (years) | 30 - 47 | 48 - 56 | 57 - 63 | 64 - 77 |
| BMI (kg/m^2^) | 22 – 26 | 27 – 28 | 29 – 30 | 31- 41 |
| TG (mg/dL) | 50 – 153 | 154 – 257 | 258 – 396 | 397 – 1324 |

BMI, Body Mass Index.

# **Supplementary Table S.3.** Pharmacological therapy used by patients

| **TREATMENT** | **N. of patients** |
| --- | --- |
| **None drug therapy** | 26 |
| **Antihypertensive drugs [n]** |  |
| ACE-inhibitor/ARB [n]  Calcium antagonist [n]  thiazide diuretic [n]  MCRA [n] | 20  5  7  2 |
| **Benzo [n]** | 5 |
| **PPI[n]** | 11 |
| **alfa-blocker [n]** | 2 |
| **Beta-blocker [n]** | 15 |
| **Antiplatelet/anticoagulant [n]** | 13 |
| **Xanthine oxidase inhibitor [n]** | 5 |
| **L-thyroxine [n]** | 6 |
| **Metformin [n]** | 3 |
| **D-vitamin [n]** | 4 |
| **adrenergic β2 receptor agonists [n]** | 2 |
| **Antihistamine [n]** | 1 |

ACE, angiotensin-converting enzyme; ARB, angiotensin receptor blocker; MCRA, mineralocorticoid receptor antagonist; PPI, proton-pump inhibitors.

# **Supplementary Table S.4.** Demographic, anamnestic and biochemical assessment in different steatosis severity groups

| **PARAMETER** | **S0** | **S1** | **S2** | **S3** |
| --- | --- | --- | --- | --- |
| **Age** [years: median; IQR] | 52 (44-55) | 57 (51-59) | 54 (43-60) | 58 (49-63) |
| **Sex** [F/M: n; %] | 3 (33.3%) / 6 (66.7%) | 2 (22.2%) / 7 (77.8%) | 4 (26.7%) / 11 (73.3%) | 8 (19.5%) / 33 (80.5%) |
| **Type 2 Diabetes Mellitus** [absence/presence: n, %] | 9 (100.0%) / 0 (0.0%) | 9 (100.0%) / 0 (0.0%) | 14 (93.3%) / 1 (6.7%) | 37 (90.2%) / 4 (9.8%) |
| **BMI** [Kg/m^2^: median; IQR] | 28.0 (27.0-28.2)* | 25.7 (23.9-26.2) | 27.1 (25.0-28.7) | 29.8 (27.7-31.8) |
| **TC** [mg/dL: median; IQR] | 225 (219-290) | 247 (213-264) | 234 (191-271) | 230 (195-265) |
| **HDL-C** [mg/dL: median; IQR] | 42 (36-47) | 43 (42-43) | 44 (35-49) | 37 (33-42) |
| **LDL-C** [mg/dL: median; IQR] | 133 (97-179) | 154 (152-183) | 164 (111-182) | 168 (115-195) |
| **TG** [mg/dL: median; IQR] | 285 (274-350) | 235 (129-343) | 214 (112-241) | 282 (172-426) |
| **Glycemia** [mg/dL: median; IQR] | 95 (92-102) | 94 (92-98) | 97 (86-103) | 99 (88-110) |
| **AST** [UI/L: median; IQR] | 27 (20-39) | 25 (20-26) | 26 (20-33) | 29 (22-40) |
| **ALT** [UI/L: median; IQR] | 27 (19-57) | 36 (22-41) | 31 (23-46) | 40 (31-54) |
| **rs1051338** [n; %] |  |  |  |  |
| c.46AA p.16TT | 8 (88.9%)** | 7 (77.8%) | 9 (60.0%) | 16 (39.0%) |
| c.46AC/CC p.16TP/PP | 1 (11.1%) | 2 (22.2%) | 6 (40.0%) | 25 (61.0%) |
| **LAL-A [**nmol/spot/h: median; IQR] | 0.44 (.37-.47) | 0.34 (.30-.44) | 0.44 (.37-.56) | 0.49 (.42-.74) |

BMI, Body Mass Index; F, female; M, male; LAL-A, Lysosomal acid lipase activity; h, hours.

*p < 0.001 Kruskal–Wallis test for BMI in S1 vs S3 and S2 vs S3 (p = 0.002 and 0.042 adjusted for Bonferroni correction, respectively)

**p = 0.016 Pearson's chi-squared test

# **Supplementary Table S.5.** Demographic, anamnestic and biochemical assessment in different fibrosis severity groups measured with SWE.

| **PARAMETER** | **F0** | **F1** | **F2** | **F3** | **F4** |
| --- | --- | --- | --- | --- | --- |
| **Age** [years: median; IQR] | 56 (45-61)* | 64 (51-73) | 65 (56-69) | 58 (58-58) | 50 (44-56) |
| **Sex** [F/M: n; %] | 13 (22.4%) / 45 (77.6%) | 1 (33.3%) / 2 (66.7%) | 2 (20.0%) / 8 (80.0%) | 0 (0.0%) / 1 (100.0%) | 1 (50.0%) / 1 (50.0%) |
| **Type 2 Diabetes Mellitus** [absence/presence: n, %] | 56 (96.6%) / 2 (3.4%) | 3 (100.0%) / 0 (0.0%) | 8 (80.0%) / 2 (20.0%) | 0 (0.0%) / 1 (100.0%) | 2 (100.0%) / 0 (0.0%) |
| **BMI** [Kg/m^2^: median; IQR] | 27.9 (25.7-29.9) | 28.8 (23.0-32.0) | 30.8 (28.4-35.2) | 29.8 (29.8-29.8) | 29.6 (27.3-31.8) |
| **TC** [mg/dL: median; IQR] | 230 (196-264) | 264 (230-265) | 258 (204-290) | 195 (195-195) | 271 (257-284) |
| **HDL-C** [mg/dL: median; IQR] | 40 (33-45) | 37 (33-43) | 38 (35-47) | 37 (37-37) | 30 (24-36) |
| **LDL-C** [mg/dL: median; IQR] | 154 (115-181) | 214 (214-214) | 196 (180-203) | 101 (101-101) | 186 (186-186) |
| **TG** [mg/dL: median; IQR] | 241 (172-350) | 394 (92-560) | 308 (114-470) | 284 (284-284) | 727 (308-1145) |
| **Glycemia** [mg/dL: median; IQR] | 95 (90-102) | 96 (80-104) | 110 (83-118) | 110 (110-110) | 94 (84-103) |
| **AST** [UI/L: median; IQR] | 26 (21-34) | 37 (26-62) | 35 (23-48) | 19 (19-19) | 22 (22-22) |
| **ALT** [UI/L: median; IQR] | 35 (24-54) | 42 (37-74) | 36 (23-54) | 21 (21-21) | 41 (41-41) |
| **rs1051338** [n; %] |  |  |  |  |  |
| c.46AA p.16TT | 34 (58.6%) | 1 (33.3%) | 3 (30.0%) | 1 (100.0%) | 1 (50.0%) |
| c.46AC/CC p.16TP/PP | 24 (41.4%) | 2 (66.7%) | 7 (70.0%) | 0 (0.0%) | 1 (50.0%) |
| **LAL-A** [nmol/spot/h: median; IQR] | 0.45 (0.37-0.60) | 0.44 (.20-.54) | 0.60 (0.43-0.74) | 0.64 (0.64-0.64) | 0.40 (0.37-0.43) |
| **CAP** [dB/m: median; IQR] | 292 (258-331) | 345 (249-345) | 343 (312-351) | 326 (326-326) | 329 (305-352) |

BMI, Body Mass Index; F, female; M, male; LAL-A, Lysosomal acid lipase activity; h, hours.

*p = 0.047 Kruskal–Wallis test for Age in F0 vs F2 (p = 0.064 adjusted for Bonferroni correction)

# **Supplementary Table S.6.** Demographic, anamnestic and biochemical assessment in different fibrosis severity groups measured with VCTE.

| **PARAMETER** | **F0-F1** | **F2** | **F3** | **F4** |
| --- | --- | --- | --- | --- |
| **Age** [years: median; IQR] | 56 (48-63) | 53 (49-62) | 47 (43-66) | 60 (50-69) |
| **Sex** [F/M: n; %] | 13 (21.3%) / 48 (78.7%) | 1 (16.7%) / 5 (83.3%) | 1 (33.3%) / 2 (66.7%) | 2 (50.0%) / 2 (50.0%) |
| **Type 2 Diabetes Mellitus** [absence/presence: n, %] | 60 (98.4%) / 1 (1.6%) | 4 (66.7%) / 2 (33.3%) | 2 (66.7%) / 1 (33.3%) | 3 (75.0%) / 1 (25.0%) |
| **BMI** [Kg/m^2^: median; IQR] | 28.0 (25.7-29.8)* | 30.2 (28.0-31.8) | 31.0 (27.7-41.4) | 31.6 (29.3-35.4) |
| **TC** [mg/dL: median; IQR] | 231 (195-264) | 256 (215-284) | 281 (204-290) | 228 (186-244) |
| **HDL-C** [mg/dL: median; IQR] | 40 (36-45)** | 34 (29-36) | 36 (33-38) | 31 (26-35) |
| **LDL-C** [mg/dL: median; IQR] | 155 (115-182) | 191 (186-195) | 148 (94-203) | 133 (115-140) |
| **TG** [mg/dL: median; IQR] | 235 (129-340)*** | 588 (308-1164) | 362 (269-1020) | 422 (371-797) |
| **Glycemia** [mg/dL: median; IQR] | 95 (89-102) | 104 (84-118) | 98 (74-110) | 104 (96-127) |
| **AST** [UI/L: median; IQR] | 26 (21-34)**** | 22 (22-23) | 48 (46-50) | 62 (29-65) |
| **ALT** [UI/L: median; IQR] | 35 (23-45) | 41 (35-55) | 39 (23-54) | 70 (46-74) |
| **rs1051338** [n; %] |  |  |  |  |
| c.46AA p.16TT | 37 (60.7%) | 2 (33.3%) | 1 (33.3%) | 0 (0.0%) |
| c.46AC/CC p.16TP/PP | 24 (39.3%) | 4 (66.7%) | 2 (66.7%) | 4 (100.0%) |
| **LAL-A** [nmol/spot/h: median; IQR] | 0.45 (0.37-0.61) | 0.64 (0.38-0.75) | 0.45 (0.39-0.58) | 0.37 (0.25-0.53) |
| **CAP** [dB/m: median; IQR] | 298 (258-345) | 317 (308-348) | 340 (302-345) | 338 (318-350) |

BMI, Body Mass Index; F, female; M, male; LAL-A, Lysosomal acid lipase activity; h, hours.

*p = 0.049 Kruskal–Wallis test for BMI in F0 vs F4 (p = 0.283 adjusted for Bonferroni correction)

**p = 0.017 Kruskal–Wallis test for HDL-C in F0 vs F4 (p = 0.073 adjusted for Bonferroni correction)

***p = 0.007 Kruskal–Wallis test for TG in F0 vs F2 and F0 vs F4 (p = 0.018 and 0.019 adjusted for Bonferroni correction, respectively)

****p = 0.015 Kruskal–Wallis test for AST in F2 vs F4 and F0 vs F4 (p = 0.093 and 0.178 adjusted for Bonferroni correction, respectively)


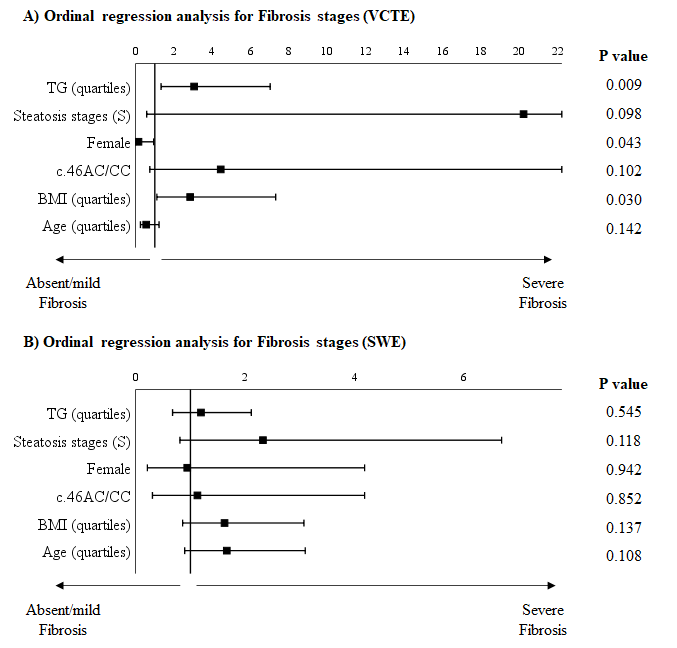


# **Supplementary Figure S.1.** Forrest-Plot of the multivariate ordinal regression analysis for the different stages of fibrosis measured with (A) VCTE and (B) SWE.

BMI, Body Mass Index; F, female; p, p-value.
